# Sample size effects on the assessment of eukaryotic diversity and community structure in aquatic sediments using high-throughput sequencing

**DOI:** 10.1038/s41598-018-30179-1

**Published:** 2018-08-06

**Authors:** Francisco J. A. Nascimento, Delphine Lallias, Holly M. Bik, Simon Creer

**Affiliations:** 1 Department of Ecology, Environment and Plant Sciences, Stockholm University, Stockholm, Sweden; 2 GABI, INRA, AgroParisTech, Université Paris Saclay, 78350 Jouy-en-Josas, France; 3 Department of Nematology, University of California Riverside, Riverside, CA USA; 4 Molecular Ecology and Fisheries Genetics Laboratory, School of Biological Sciences, Bangor University, LL57 2UW Bangor, United Kingdom

## Abstract

Understanding how biodiversity changes in time and space is vital to assess the effects of environmental change on benthic ecosystems. Due to the limitations of morphological methods, there has been a rapid expansion in the application of high-throughput sequencing methods to study benthic eukaryotic communities. However, the effect of sample size and small-scale spatial variation on the assessment of benthic eukaryotic diversity is still not well understood. Here, we investigate the effect of different sample volumes in the genetic assessment of benthic metazoan and non-metazoan eukaryotic community composition. Accordingly, DNA was extracted from five different cumulative sediment volumes comprising 100% of the top 2 cm of five benthic sampling cores, and used as template for Ilumina MiSeq sequencing of 18 S rRNA amplicons. Sample volumes strongly impacted diversity metrics for both metazoans and non-metazoan eukaryotes. Beta-diversity of treatments using smaller sample volumes was significantly different from the beta-diversity of the 100% sampled area. Overall our findings indicate that sample volumes of 0.2 g (1% of the sampled area) are insufficient to account for spatial heterogeneity at small spatial scales, and that relatively large percentages of sediment core samples are needed for obtaining robust diversity measurement of both metazoan and non-metazoan eukaryotes.

## Introduction

Predicting how benthic diversity and community structure respond to environmental change will continue to be an important challenge for aquatic ecology. Such a task is dependent on fast and reliable methods to assess diversity, particularly in ecosystems like soft-sediment habitats, where most eukaryotic diversity is comprised of microscopic communities^1,2^ crucial to a number of important ecosystem processes^3–6^. Despite their demonstrable contributions to important ecological functions, the levels of eukaryotic and, in particular, metazoan diversity are not well known, due to a number of practical limitations involved in the taxonomic and ecological investigations of microscopic eukaryotic communities^7^. Among these, the level of time and financial resources necessary to cover such broad taxonomic scales are perhaps the most important^1,7,8^.

The application of high-throughput sequencing (HTS) to the study of benthic eukaryotes has the potential to significantly advance our knowledge of interstitial eukaryotic diversity^8–10^. HTS techniques can be applied for species identification from samples where organisms have been separated and isolated from the sediment/soil matrix or water before the analysis^10^, or from environmental DNA (eDNA). Environmental DNA is here defined as the genetic material attained from samples (soil, sediment, water) irrespective of whether the DNA is intracellular or extracellular, or associated with living or dead biomass^11^.

Establishing an appropriate and standardized sampling strategy is critical to the use of HTS for studies of benthic eukaryotic biodiversity, particularly since the application of HTS techniques to benthic ecology questions is relatively recent^10,12,13^. Sediment ecosystems often include a matrix of different particles and aggregates that form highly complex and heterogeneous physical environments at both local (e.g. biogenic structures like feeding pits or fecal mounds create heterogeneity at a centimeter scale) and at regional spatial scales where benthic habitats encompass different habitats such as sandy or soft-sediments kilometres apart^14,15^. At finer scales, heterogeneity and spatial structure can hinder the detection of ecological patterns and processes at ecosystem scales^15,16^. In particular, the large number of niches contained within small spaces in sediments^14^ leads to a heterogeneous distribution of eukaryotic microbial communities^16,17^. In addition, DNA from aquatic organisms is itself often distributed heterogeneously^18^ and in benthic habitats it can adsorb more or less efficiently to different minerals^19^, creating additional spatial heterogeneity. This DNA adsorption capacity can differ greatly between sediment types, with clay minerals typically having a hundred-times higher adsorption capacity than sandy sediments^20^. The study of biodiversity using DNA sampling is therefore dependent on several factors like the concentration of DNA in the sampled habitat, DNA capture and extraction efficiency and sample interference (e.g. inhibition)^21^.

Common strategies to deal with small scale heterogeneity include the collection of large samples (often unpractical and costly) or several smaller samples that are pooled, homogenized and from which a smaller sub-sample is taken and analysed, that is considered to be representative of the entire mix^17^. Lanzén *et al*.^22^ showed that pooling increasing numbers of DNA extraction replicates significantly improved eukaryotic diversity estimates for both protists and metazoans in marine sediments. Conversely, recent works using HTS for environmental monitoring of ciliates indicate that replicates from small sediment samples are similar to each other^23^. The strategy of extracting DNA directly from small homogenised samples of sediment without subsequent isolation of organisms from the sediment matrix is often utilized in works employing HTS for the assessment of benthic microeukaryotic diversity^24–26^, although such studies often emphasize diversity patterns in single-celled eukaryotes (e.g. protists) (See Supplementary Table S1 for examples of previous work that assessed eukaryotic diversity in aquatic sediments using high-throughput sequencing)^27–36^. Additionally, a number of works have successfully employed eRNA in environmental monitoring assessments of marine eukaryotes^23,37–40^. Even though the sampling of eRNA presents additional storage and preservation challenges, it can depict accurately the active fraction of interstitial communities^37,38,41^. Nevertheless, eDNA has a higher persistence than eRNA and has been shown to capture more representative measures of interstitial biodiversity than eRNA^41^.

However, how much such strategies can tell us about sediment metazoan diversity is not clear as small samples do not seem to be effective in capturing metazoan meiofauna diversity. Meiofaunal organisms are physically larger (38 µm-1 mm in size), have patchy distributions in space and time, and most likely have lower effective population sizes compared to bacterial and protist populations^14^. As such, when targeting meiofauna diversity, isolation from larger sediment samples is recommended^12^. However, meiofauna studies typically employ decantation or density extraction and sieving in order to concentrate metazoan organisms and physically separate them from sediment grains. Ecologically important organisms such as single-celled microeukaryotes and taxa <38 µm in size are lost during these physical extraction/isolation protocols. There are obvious advantages in the use of the same sample for HTS diversity assessments of multiple communities of different size fractions (eg. protists, bacteria and meiofauna)^39^. Only by measuring these metrics in the same sample will we be able to capture and understand the interspecific interactions occurring in small spatial scales and how they affect sediment diversity.

As such, one important methodological question that needs to be addressed for benthic HTS is what sample volume from a homogenized mixture can adequately represent the diversity and community structure of both microeukaryotes and metazoans in the sediment. Sample size can affect the measurement and interpretation of biodiversity metrics, particularly in species rich ecosystems^42^. Insufficient sample sizes are responsible for non-trivial biases on estimations of beta-diversity that are hard to predict^42^. Since diversity metrics are essential tools for benthic ecological research, with the emergence of HTS, it is vital to understand how sample volumes influences biodiversity metrics in aquatic sediments.

Soil microbial ecologists have found sample volume to have an effect on the patterns of soil microbial biomass^43^ and on how accurately microbial community structure and diversity is assessed^44–46^. For example, soil volumes of 10 g were related to higher richness, diversity and evenness^46^ and with lower variability between replicates than smaller sample volumes^44^ (see Supplementary Table S2 for an overview of studies that investigated effects of sample volumes on diversity assessments). Nevertheless, few studies have looked at the effects of sample volumes using HTS in aquatic sediments.

Therefore, we address in our study an important question in biodiversity assessments of marine sediments using HTS: what percentage of a core sampling unit is a representative and appropriate sample volume for a reliable measurement of both micro-eukaryotic and metazoan diversity and associated community structure?

Consequently, we devised a novel experimental design that analysed metazoan and non-metazoan eukaryotic diversity and community composition in replicated samples of five different sediment volumes (0.2, 4, 6, 8 and 10 g) that together comprised 100% surface area of a sampling core. Sampling cores are generally considered the best quantitative sampling technique for benthic communities in marine clay and silt sediments^47^ and here we employed a sampling core commonly used in both field and experimental studies of benthic ecology^3–5^. Following environmental DNA extractions, we sequenced the V4 region of the 18 S nuclear small subunit (nSSU) ribosomal RNA (rRNA) gene from these different sediment quantities and used ca. 400 base pair amplicons to assess the impact of sample volume on alpha and beta-diversity measurements. Our findings contribute to the development of consistent protocols to facilitate the comparison among ecological studies on benthic eukaryotic communities.

## Results

### HTS data output

Community analysis was performed on both the 96% and the 99% OTUs and produced similar results. For conciseness, we present the 96% OTU data here and the 99% OTU data in Supplementary Information (See Supplement Table S3 for statistics regarding the 99% OTUs).

In total the Illumina Miseq sequencing of eukaryotic 18 S rRNA amplicons yielded 10 320 000 raw paired-end reads from 25 samples from which 7 776 371 quality-filtered reads were assembled. After chimera removal there was an average of 305 997 sequences per sample (min-168 672; max- 572 017; Supplement Table S4). Clustering at 96% OTU similarity produced 10 220 OTUs assigned to domain Eukaryota after singleton and doubleton removal, of which 4870 were non-metazoan eukaryotes, 870 OTUs were from metazoan taxonomic groups and 4480 could not be assigned to Order. OTU accumulation plots for Non-metazoan Eukaryotes and Metazoans are presented in Supplementary Information (Supplementary Fig. S1).

#### Alpha diversity differences with sample volume

**Figure 1:**
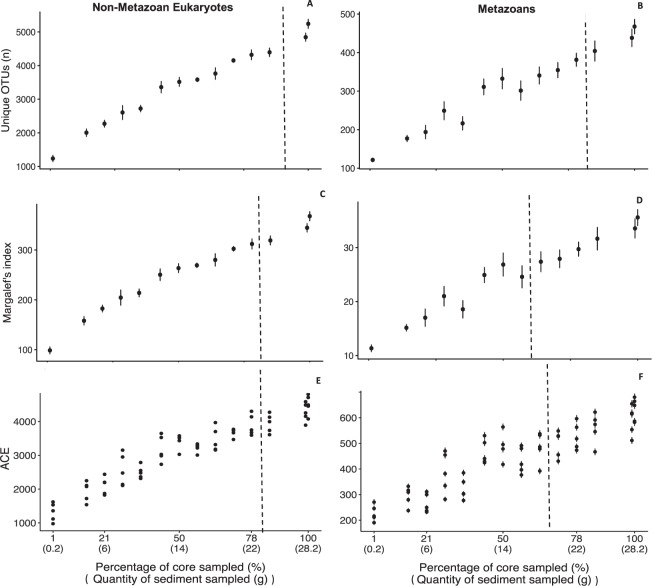
Diversity measurements for Non-Metazoan Eukaryotes (**A**,**C**,**E**) and Metazoans (**B**,**D**,**F**) in the different treatments. Figure panels show number of unique OTUs (**A**,**B**), Margalef Diversity index (**C**,**D**) and ACE index (**E**,**F**). Dotted line indicates the cutoff point after which the differences to the 100% treatment stop being significant (see Table 1). Diversity measurements were performed on transformed data. Data represents 96% OTUs.

The number of unique OTUs was lowest in the 1% of the total sampled area (0.2 g) with an average of 1238 and 121 OTUs for non-metazoan eukaryotes and metazoans, respectively, and increased with sample volume to a maximum of 5243 and 468 eukaryotic and metazoan OTUs in the treatment with 100% of the total sample area (28.2 g) (Fig. 1A,B). The difference in the number of unique non-metazoan OTUs between the 100% treatment and all other treatments was significant for all sample volumes below 85% of the total area sampled (24 g), but not for any remaining sample volumes above that value (Table 1). A similar pattern was seen for the number of unique metazoan OTUs but with a lower threshold, at 64% of the total area sampled (18 g) (Table 1). There was also a significant effect of sample volume on diversity for both non-metazoan eukaryotes and metazoans (Fig. 1C–E, Table 1). Pairwise comparisons between the treatment comprising 100% of the sample area (28.2 g) and all other treatments show that the differences in Margalef and ACE for non-metazoan eukaryotes were significant for all sample volumes below 71% (20 g) and 78% (22 g), respectively, but not for larger sample volumes (Table 1). For metazoans that cut-off was at 57% of the total area sampled (16 g) for Margalef and 63% (18 g) for ACE index (Table 1), respectively.

**Table 1:** Summary of statistical tests comparing the different treatments with the treatment comprising 100% of the sampled area (96% OTUs).

	Community	ANOVA F statistics	ANOVA p value ANOVA	Lowest % of core sampled significantly different from the 100% treatment
Percentage of metazoan taxa	NA	4.1	<0.001	1% (0.2 g)
Number of unique OTUs	Non-Metazoan Eukaryotes	67	<0.001	85% (24 g)
	Metazoans	23	<0.001	78% (22 g)
Margalef index	Non-Metazoan Eukaryotes	57	<0.001	71% (20 g)
	Metazoans	17	<0.001	57% (16 g)
ACE richness	Non-Metazoan Eukaryotes	51	<0.001	78% (22 g)
	Metazoans	19	<0.001	63% (18 g)
		**PERMANOVA F Statistics**	**PERMANOVA p value**	**Lowest % of core sampled significantly different from the 100% treatment**
Beta-Diversity	Non-Metazoan Eukaryotes	2.5	<0.001	43% (12 g)
	Metazoans	1.9	<0.001	71% (20 g)

One-way parametric analysis of variance ANOVA was used to test for the effect of sample volume. P values smaller than 0.01 indicate a significant effect of sample volume. Pairwise comparisons between the 100% treatment and the other 13 treatments were performed with the Tukey HSD test. Effects of sample volumes on beta-diversity differences among treatments were tested with PERMANOVA. Differences between the 100% treatment and the other 13 treatments for beta-diversity was tested with PERMDISP. The treatment with the highest sample volume still different from the treatment comprising 100% of the sampled area in each of the variable is presented in the last column of the table.

#### Beta-diversity differences with sample volume

**Figure 2:**
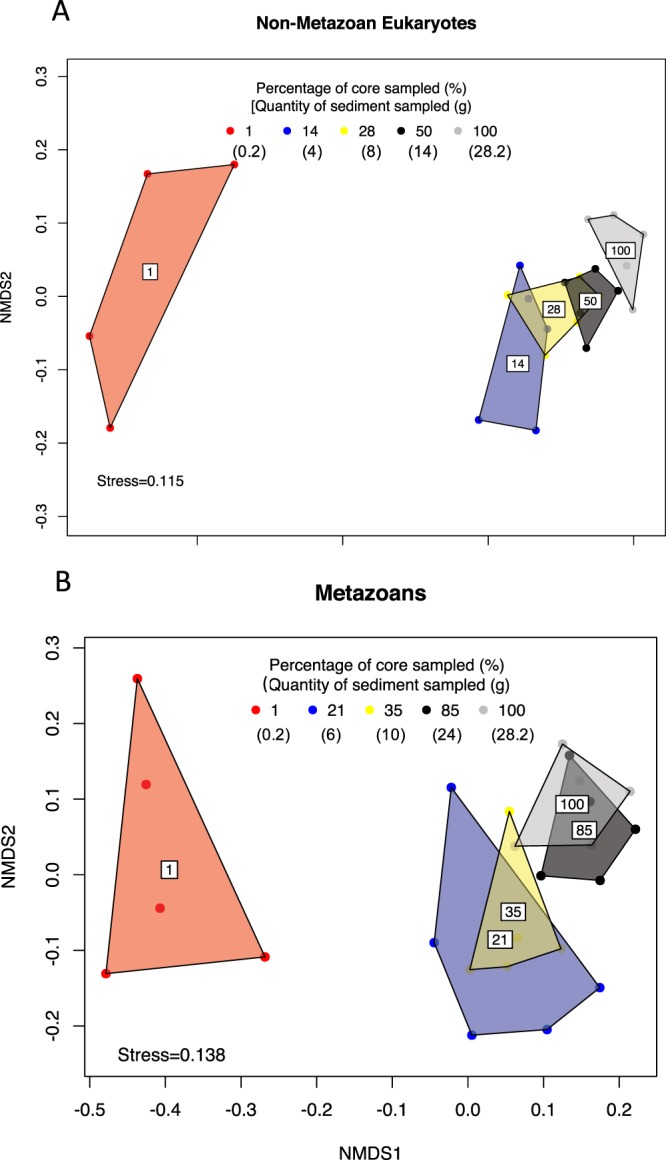
Simplified non-metric multidimensional scaling (NMDS) analysis of the Sørensen beta-diversity matrix based on transformed OTU table for Non-Metazoan Eukaryotes (**A**) and Metazoans (**B**). Differences between the different treatments and the 100% treatment stopped being significant at 43% of sediment analyzed for Non-Metazoan Eukaryotes and in the 78% treatment for Metazoans (PERMANOVA, PERMDISP, see Table 1). For better visualization, only the treatment comprising 100% of the sampled volume, 3 treatments significantly different from the 100% treatment (1, 14, 28% for Non-Metazoan Eukaryotes and 1, 21 and 35% for Metazoans), and one treatment not significantly different from the 100% treatment (50% for Non-Metazoan Eukaryotes and 85% for Metazoans) are here shown. Data represents 96% OTUS.

**Figure 3:**
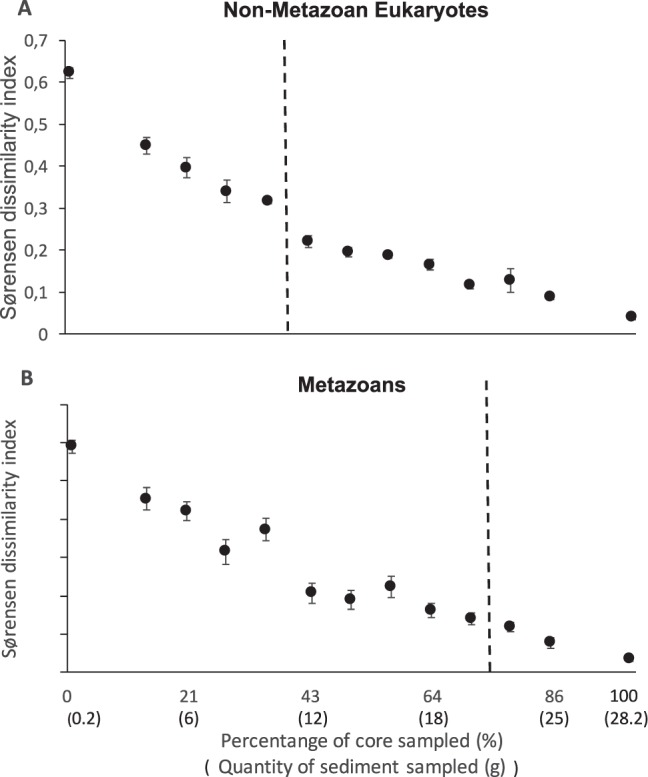
Average Sørensen dissimilarity index in each treatment in relation to the treatment where 100% of the sampled area was analyzed ((**A**)-Non-Metazoan Eukaryotes; (**B**)- Metazoans). Circles represent average dissimilarity in relation to the 100% treatment and error bars SE. Dotted line indicates the cutoff point after which the differences to the 100% treatment stop being significant (see Table 1). Sørensen dissimilarity index was calculated using transformed OTU data. Data represents 96% OTUS.

Figure 2 shows a simplified NMDS ordination of the non-metazoan eukaryotic (Fig. 2A) and metazoan (Fig. 2B) community composition. An NMDS and PCoA plot with all treatments for the 96% OTUs can be seen in the Supplementary Information (Supplement Fig. S2-A,S2-B, for non-metazoan eukaryotes and metazoan NMDS, and Supplement Fig. S3-A,S3-B for PCoA ordination, respectively). PERMANOVA analysis showed a significant effect of sample volume in both non-metazoan eukaryotic and metazoan community beta-diversity (Table 1). PERMDISP revealed significant overall differences among treatments. Non-metazoan community composition of the 100% treatment (28.2 g) was different from all sample volumes below 43% of the total sampled area (12 g) but not significantly different from any of the sample volumes equal or larger than this value (Table 1). The differences in beta-diversity between the treatment comprising 100% of the area sampled (28.2 g) and all other sample volumes are not significant for the 71% treatment (20 g) and above. These dissimilarities among treatments in relation to the 100% of the area sampled decreased clearly with increased sample volume (Fig. 3). Data analysis with 99% OTUs presented similar results (see Supplement Table S3 for statistics, Supplement Fig. S4 for simplified NMDS and Fig. S5 for PCoA).

Unsurprisingly, differences between the 100% treatment and smaller sample volumes (0.2 g, 4 g, 8 g, 10 g and 12 g) were mostly driven by nestedness, while turnover was more influential in explaining overall beta-diversity differences between larger sample volumes and the 100% treatment. This pattern was seen for both Non-Metazoan Eukaryotes and for Metazoans (Supplement Fig. S6A,B, respectively).

#### Additional sample volumes and read combination

**Figure 4:**
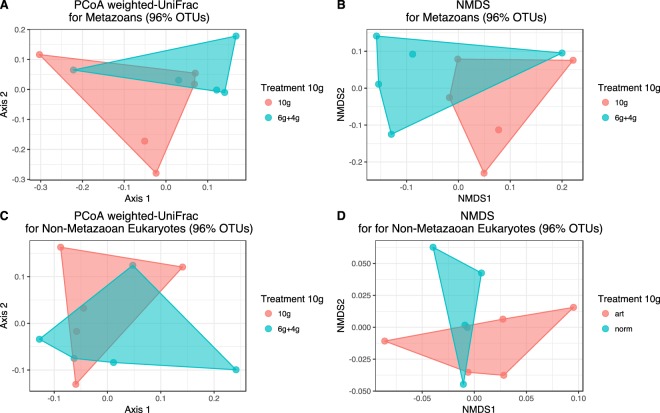
PCoA (**A**,**C**) and NMDS (**B**,**D**) ordinations comparing two different strategies for obtaining 10 g with the sample volumes used in this study: 6 + 4 g (in blue) vs 10 g (in red). No statistical differences were seen between the two treatments (PERMANOVA, p = 0.24 and p = 0.65). Data represents 96% OTUS.

**Figure 5:**
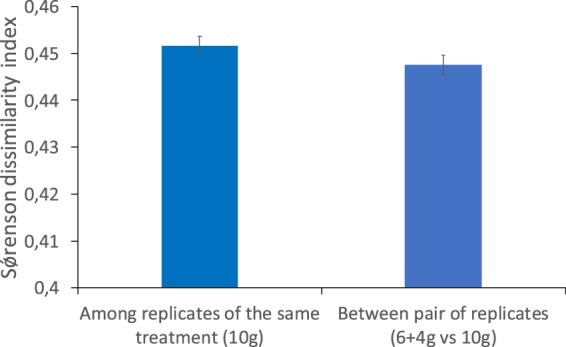
Average Sørensen dissimilarity index among replicates of the 10 g and distance between 10 g vs 6 g + 4 g of the same replicate (based on data for Metazoans). Data represents 96% OTUS.

Combining reads of different sample volumes had no effect on beta-diversity measurements. As it is possible to see in both the NMDS plot and in the weighted PCoA (Fig. 4), there is considerable overlap between the two ways of analysing sample volume of 10 g here tested (6 g + 4 g vs 10 g) for both metazoan and non-metazoan eukaryotic beta-diversity. We found no statistical differences in beta-diversity between these two ways of obtaining 10 g of sediment (PERMANOVA, p = 0.24 and p = 0.65 for metazoans and non-metazoan eukaryotes, respectively). In fact, Sørensens dissimilarity among the 5 replicates of the 10 g treatment is higher than the average dissimilarity between the pairs of 6 g + 4 g and 10 g for each replicate (Fig. 5). Both these two analyses suggest that we obtain very similar representations of beta-diversity if we pool 6 g + 4 g or if we analyse 10 g directly. Additionally, we performed a similar test to investigate whether there were significant differences in beta-diversity if we pooled 8 + 6 g vs 10 + 4 g to get 14 g and 8 g + g6 + 4 g vs 1g0 + 8 g to get 18 g. We found the same pattern as mentioned above for 10 g vs 6 + 4 g (Supplement Fig. S7). No statistical differences were found in beta-diversity (PERMANOVA, p = 0.53 and p = 0.92 for 14 g and 18 g, respectively) and higher dissimilarity between the replicates of the same pooling combination than the replicates obtained with different pooling combinations (Supplement Fig. S8). Taken together, these results show clearly that there is no effect of pooling samples and that the differences seen in our study are indeed due to sample volume.

Our data analysis was performed with relative OTU abundances, suggested by McMurdie & Holmes^48^ as an appropriate and effective alternative to rarefying. However, the same authors warn that artefacts due to varying sampling depths can persist even when using relative OTU abundance^48^. Since we cumulated reads numbers from 0.2–10 g volumes to represent up to 100% of the cores, it is possible that there was an over-inflation of rare taxa in the larger sample volumes that would affect our diversity metrics. However, as discussed above and shown in Figs 4 and 5 and Figs S7 and S8 in Supplementary Information, combining reads of different sample volumes had no significant effects on beta-diversity assessment. Furthermore, an analysis with a dataset trimmed to contain only the 30 most abundant non-metazoan eukaryotic taxa in each sample volume, produced similar results to the whole data set in terms of the effect of sample size on beta-diversity measurements (PERMANOVA, F = 8.0, p = 0.001, NMDS plots presented in Fig. S11 Supplementary Information). Differences in beta-diversity between the 100% treatment and the six smaller samples sizes (0.2 g, 4 g, 6 g, 8 g, 10 g and 12 g) tested with PERMDISP were all significant, but not for the remaining sample sizes above 12 g.

#### Taxonomic composition

**Figure 6:**
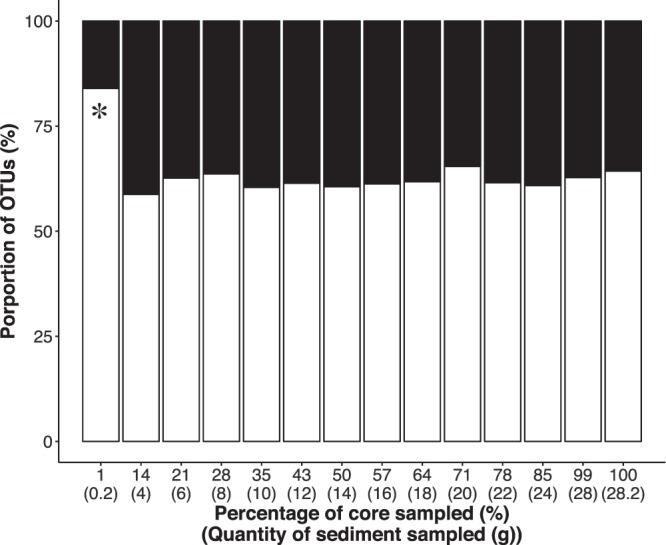
Differences among treatments in proportion of Metazoan (full bar) vs Non-Metazoan Eukaryotes (empty bars). Asterisk indicates a significant difference among treatments in proportion of metazoan OTUs. These proportions represent transformed OTU data, from 96% OTUs.

**Figure 7:**
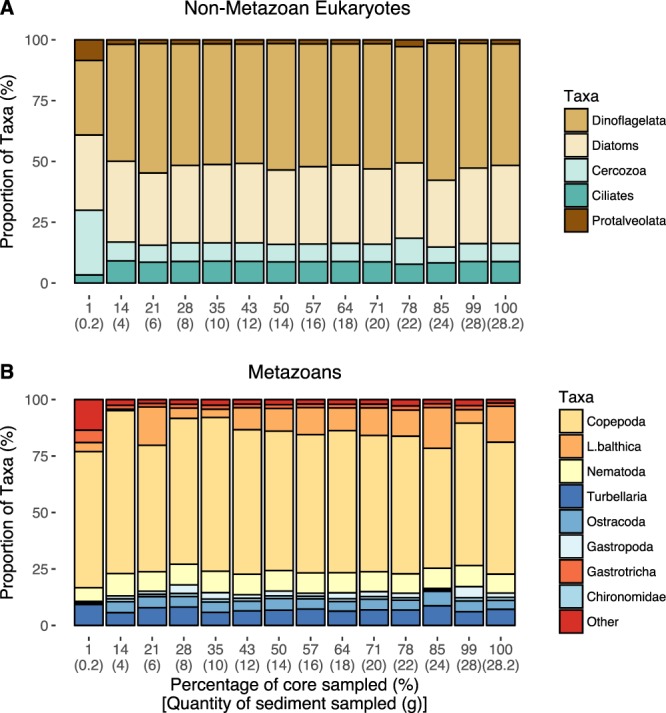
Proportion of the most abundant taxonomic groups in each treatment: (**A**) Top five most abundant Non-Metazoan Eukaryotes in each treatment; (**B**) Top nine most abundant Metazoan taxa in each treatment. Data represents 96% OTUs.

The percentage of OTUs belonging to metazoan groups was different among sample volumes (Fig. 6), with the smallest sample volume (1% of the total sampled area, corresponding to 0.2 g) containing significantly less metazoan OTUs than all the other treatments (ANOVA, p < 0.001, Table 1). No additional significant differences among other sample volumes in metazoan vs non-metazoan eukaryote taxa were detected. Generally, the most abundant non-metazoan eukaryotic groups were the dinoflagellates, followed by diatoms and ciliates (Fig. 7A and Supplement Fig. S9-A). Copepod OTUs dominated the metazoan dataset comprising more that 70% of the relative abundance. Bivalvia (mostly the clam *Limecola balthica*) and nematodes OTUs were the other most frequent metazoan groups in our study (Fig. 7B and Supplement Fig. S9-B).

## Discussion

### Effects of sample volume on both alpha and beta-diversity

Our results show clearly that sample volume affected all non-metazoan eukaryotic and metazoan diversity metrics investigated here. Sample volume had a significant effect on percentage of metazoan OTUs, number of unique OTUs, Margalef and ACE diversity indexes both for non-metazoan eukaryotes and metazoans (Fig. 1, Table 1).

These results are in accordance with previous studies that found sample volume to have a significant influence when investigating the diversity and community composition of soil bacteria^17,44^. In addition, Penton *et al*.^46^ using HTS to look at soil fungal and bacterial communities found that alpha diversity, richness and evenness to be larger with larger sample volumes showing a similar pattern to our results. However, related effects of sample volume on richness and alpha diversity were not seen in marine meiofauna in Brannock and Halanych^12^. Although these authors compared alpha diversity metrics among samples of different volumes, only two replicates were employed, which might account for the different results. Brannock & Hananych^12^ also used a different set of primers on fully marine sediments that generally include more diverse communities than soft-sediment brackish habitats like the Baltic Sea^14^.

Sample volume was also a significant factor when analysing beta-diversity for both non-metazoan eukaryotes and metazoans, with the smallest sample volume clearly separately from all other groups (Fig. 2). Our results indicate that dissimilarity among treatments in relation to the 100% treatment decreases with sample volume (Fig. 3), showing that sample volume is an important factor when assessing sediment beta-diversity. Indeed, the larger the sample volume, the more animals are captured and the more similar the samples are to the 100% treatment. Sample volume effects on beta-diversity and community composition were seen on different taxonomic groups from other studies, including bacteria, eukaryote and metazoans in both terrestrial and aquatic ecosystems^12,17,44,46^. While the effects of sample volume on beta-diversity were significant in our metazoan and non-metazoan eukaryotes datasets (Fig. 2, Table 1), there were important differences between these two taxonomic groups. Our results suggest that the analysis of a much larger percentage of the core is needed for metazoans (78%) than for non-metazoan eukaryotes (43%) in order to achieve a representative picture of the real beta-diversity, indicating a higher degree of patchiness for the metazoan community. Such small spatial scale patchiness has also been reported for small benthic metazoans like meiofauna^12,14^ as these animals tend to cluster in microsites rich in organic matter content^14,49^. A common strategy in studies focusing on meiofauna community structure is to isolate individuals from large sediment sample sizes by decantation or density extraction in a 38–45 µm sieve before DNA extraction. Decantation concentrates living individuals from a large sample size into a smaller volume and reduces the quantity of extracellular DNA, DNA from dead/decaying, resting stages and the quantity of PCR-inhibiting humic acids in the analysed sample. Such a strategy is recommended by previous studies for HTS assessments of meiofauna diversity^12^. However, decantation will necessarily involve the loss of information from protists and eukaryotes smaller than 38 μm and soft-bodied animals in the meiofauna fraction. For studies aiming at assessing biodiversity of both the larger meiofauna and microeukaryotes, the effects of decantation must therefore be considered. Combining multiple sub-samples for microbial eukaryotes, with sieving and isolation of the larger meiofauna from the remaining sediment sample, are a feasible alternative strategy when aiming for simultaneous recovery of benthic taxa representing different size fractions and trophic levels^50^. The extracted DNA for each size fraction could then be PCR amplified and bioinformatically pooled after sequencing for effective diversity assessments. In addition, this strategy is useful in studies using sampling equipment with larger surface areas such as Van Veen grab sampler. This sampling equipment has a total surface area considerably larger than the core used in our study and is often used in environmental monitoring studies that employ HTS in sediment surveys of diversity (e.g.^23,40,41,51–53^). As such, sampling volumes equivalent to for example 43% and 78% of the surface area of a Van Veen grab sampler will not be realistic for logistical reasons and strategies that employ combination of multiple sub-samples for microeukaryotes together with animal isolation for metazoans are appropriate^23,39–41,51–53^. Studies using Van Veen grab to sample sediment for HTS assessments of eukaryotic diversity often randomly collect 1.5–2 g of sediment from across the top 1 cm surface layer of the grab to address the issue of micro-scale patchiness^23,39–41,51–53^. Unfortunately, we were not able to test in our experiment sample volumes of 1.5–2 g, as the two DNA extraction kits here used that contained the same reagents, were optimized for sample volumes not smaller that 4–5 g (PowerMax® Soil DNA Isolation Kit) or larger than 0.2 g (PowerSoil® DNA Isolation Kit). To test a sample volume of 1.5–2 g efficiently we would need to use a separate extraction kit with different protocols and reagents, making the comparison with remaining sample volumes difficult. Testing these often used sample volumes in future studies would provide valuable information.

#### Community composition

We found a high diversity of eukaryotes in brackish sediments of the Baltic Sea. In terms of relative OTU abundance (Fig. 6 and Supplement Fig. S6) the Copepoda was the most represented group, despite nematode being generally the most abundant taxon in metazoan datasets for brackish and marine sediments^7,12,54^. It is possible that the higher prevalence of copepod OTUs here found is related to: (a) the degenerate nature of the eukaryotic primers sets used in our study^10,55^, (b) high biomass of copepod tissue that was potentially differentially amplified^56^ and (c) the extraction strategy used in our study designed to capture total (both intra- and extracellular) DNA. As such, and taking into account the size of our amplicons (~400 bp), our sample likely reflects DNA from contemporary and recently inhabiting organisms, in addition to DNA from planktonic organisms that has settled to the benthos. Copepods species such as *Acartia bifilosa* and *Arcatia tonsa* found in our dataset deploy sediment resting stages^57^, thereby suggesting marine snow as the likely source of copepod eDNA. Similarly, Guardiola *et al*.^37^ recovered more Arthropoda diversity compared to nematodes in 18 S nSSU HTS analyses from samples obtained from deep sea canyons where eDNA was extracted directly from sediment. Nevertheless, our study detected 194 unique nematode OTUs suggesting that we did manage to effectively sample metazoan diversity (Supplement Fig. S10).

#### Concluding remarks

Collectively our work indicates that small spatial variability, even within a sampling unit, is a factor that needs to be taken into account in studies investigating eukaryotic and metazoan diversity in aquatic sediments. To achieve a representative assessment of the beta-diversity for both these communities it is necessary to sample a large percentage of the sampling unit (78% or 22 g). We saw similar pattern for alpha-diversity metrics (Table 1, Fig. 3B,D,F) but the different estimates of richness for the 100% treatment stop being significant at around 60% of the sampling unit. As such, if one is targeting solely metazoans it is probably more efficient to first separate the metazoan community from the sediment particles using elutriate or density extraction techniques^4,14^ before DNA extraction as suggested by Brannock & Halanych^12^. Furthermore, when larger sampling units are to be used (e.g. Van Veen grabs) to simultaneously sample multiple size fractions of sediment communities, combining multiple sediment subsamples for microeukaryotes with sediment sieving and isolation of larger taxa has been shown to be effective^23,41^

Nevertheless, it is clear from our study that small sample volumes of 0.2 g (1% of the sampled area), often used in benthic surveys to address sediment benthic diversity are not large enough to account for spatial heterogeneity, even at small spatial scales, and measure reliably both alpha and beta-diversity in non-metazoan eukaryotic communities. In contrast to other studies with marine sediments we did find a strong effect of sample volumes on alpha-diversity metrics for non-metazoan eukaryotes indicating a need for the use of larger sample volumes. In addition, our data suggest that approximately 14 g of homogenized sediment in our study needs to be sampled to achieve an adequate beta-diversity measurement.

The larger sample volumes here discussed seem to capture adequately the complex heterogeneous matrix of microhabitats that soft sediments provide for both micro metazoans and eukaryotes. In addition, our results are based on the use of a sampling core of one size, albeit a common one used in studies of micrometazoans^3–5^ with sediment from one location in a coastal muddy soft-sediment habitat. It would be valuable to test these recommendations with additional sample volumes commonly used in field studies, and different locations with sediment of different types collected with sampling equipment of lager sizes.

Our results are relevant for both field and experimental studies in marine systems, but the present investigation did not aim to replicate current sampling strategies used in ongoing benthic monitoring initiatives (e.g. in terms of number of replicates per site and overall number of sample sites). However, our findings indicate that DNA extraction volume can significantly impact biodiversity estimates derived from metabarcoding approaches, and this should be taken into account when designing future DNA-based environmental monitoring strategies.

Our study underlines the need for a robust standardization of methods to investigate the diversity of eukaryotic communities and allow for cross-study comparisons, particularly taking into account the foreseeable future where DNA techniques will become more important in efforts to assess the dynamics of biodiversity in aquatic sediments^58^.

## Material and Methods

### Sample collection

Silt muddy sediment was collected with five handheld Perspex sediment cores taken approximately 40–50 cm from each other from a depth of 15 m at a temperature of 5 °C and salinity of 6 psu in the Stockholm archipelago (58°50′N, 17°32′E) in the northern Baltic Sea, Sweden. The handheld sampling units were 44 mm diameter with a surface area of 15 cm^2^, a size which has been found to be appropriate for sampling of microbial benthic metazoans such as meiofauna^59^. After collection, the sediment cores were transported to Stockholm University and placed in a thermoconstant room at 5 ± 1 °C overnight. Previous studies report that >95% of the diversity of Baltic Sea metazoan diversity is found within the first centimetre of sediment^60^. As such, on the following day, the top 2 centimetres of sediment from each replicate sediment core were sliced, homogenized, weighed and divided into five sample volumes: 0.2 g, 4 g, 6 g, 8 g, and the remaining amount of sediment that varied between 10–11 g, corresponding to 1, 14, 21, 28 and 36% of the top 2 centimetres of each core. In this way, all the sediment from the top 2 cm of each of the five replicates was analysed, with the experimental design facilitating the investigation of the effect of sample volume in the composite core. The sediment samples were then placed in 50 mL Falcon tubes and kept at −20 °C until DNA extraction, with the exception of the 0.2 g samples that were placed in 1.5 mL Eppendorf tubes. The sample volumes were chosen taking into account the practical limitations of existing DNA extraction commercial kits and the need to have a sufficiently large number of different sediment quantities to test the effect of sample volume while looking at the whole surface area of the sampling unit at an affordable cost.

### Sample preparation and sequencing

For the 0.2 g samples, DNA was extracted with the PowerSoil® DNA Isolation Kit (MOBIO, Cat#12888), following the protocol instructions. The PowerMax® Soil DNA Isolation Kit (MOBIO, Cat#12988) was used for the remaining sample volumes (4 g, 6 g, 8 g and 10 g). The protocol, methodology and chemical reagents used in the different kits are identical, the only difference being the amount of sediment each kit can process^12^. After DNA extraction, samples were frozen at −20 °C in 100 μL (0.2 g samples) and 3 mL (all other sample volumes of C6 solution (10 mM Tris)). Following this, 100 μL of each DNA extract was purified using PowerClean^®^ Pro DNA Clean-Up Kit (MOBIO, Cat# 12997–50) and stored in 100 μL of C5 (10 mM tris) solution at −20 °C. DNA extraction from different sample volumes yielded DNA extracts of different concentration ranging from 62.6 to 10.1 ng/μL. Before PCR amplification all DNA extracts were standardized to a concentration of 10 ng/μL.

The highly conservative metabarcoding primers TAReuk454FWD1 (5′-CCAGCA(G/C)C(C/T)GCGGTAATTCC-3′) and TAReukREV3 (5′-ACTTTCGTTCTTGAT(C/T)(A/G)A-3′)^61^ and Pfu DNA polymerase (Promega, Southampton, UK) were used to PCR amplify the 18 S nSSU gene region, yielding fragments between 365–410 bp not including adaptors or barcodes. Each sample volume from the five replicate sampling cores were amplified in triplicates. These triplicates were then pooled, dual-barcoded with Nextera XT index primers following Bista *et al*.^62^ and visualized by gel electrophoresis. These tagged amplicons were later purified with the Agencourt AMPure XP PCR Purification kit (Beckman Coulter), quantified with Qubit (Invitrogen, USA) and pooled in equimolar quantities. The purified amplicons were then sequenced in both directions on an Illumina MiSeq platform at National Genomics Infrastructure (NGI-Stockholm, Sweden) as a single pool comprised of the 25 different samples with 25 unique index primer combinations (i.e., five sample volumes – 0.2 g, 4 g, 6 g, 8 g, and 10 g – from 5 different sampling core replicates).

### Bioinformatics

Amplicon reads were demultiplexed by NGI and raw demultiplexed reads were used in the initial data processing and quality-filtering was carried out in QIIME 1.8^63^. Raw Illumina reads were first subjected to read pairing (merging Read 1 and Read 2) using the join_paired_ends.py in QIIME, followed by a demultiplexing step using the multiple_split_libraries_fastq.py script which split samples according to dual-index barcodes and also performed quality-filtering of raw reads. Remaining forward and reverse PCR primer sequences were subsequently removed from Illumina reads using Trimmomatic version 0.32^64^. Processed reads were concatenated into a single file, and then subjected to open-reference OTU picking with Uclust using the pick_open_reference_otus.py script in QIIME (with 10% subsampling, no prefiltering, and reverse strand match enabled). Singleton and doubleton OTUs were discarded from the resulting OTU tables (retaining only OTUs with >2 reads). Two independent OTU picking workflows were carried out, using pairwise cutoffs at 96% and 99% sequence identify (hereby referred to as 96% and 99% OTUs). Taxonomy was assigned to representative OTU sequences using the RDP Classifier^65^ in QIIME (assign_taxonomy.py), using the SILVA 119 release as a reference database^66^. OTU representative sequences were aligned using PYNAST^67^ in align_seqs.py and used to construct a Phylogenetic tree using FastTree^68^ in make_phylogeny.py. All bioinformatics commands, OTU tables, and quality-filtered Illumina reads have been posted on FigShare (doi: 10.6084/m9.figshare.4993667).

### Community analyses

The number of reads for the five sample volumes (0.2 g, 4 g, 6 g, 8 g and 10 g) of the same replicate were then added in all possible combinations to produce nine additional sample volumes (12 g, 14 g, 16 g, 18 g, 20 g, 22 g, 24 g, 28 g and the complete 28.2 g) for the five replicate cores, corresponding to 43, 50, 57, 64, 71, 78, 85, 99 and 100% of the sediment in the top 2 cm of each core (see Supplement Table S5). This data set was imported into R v 3.3.2 (R Core Team 2016) and analysed using the *phyloseq*^69^ and *vegan*^70^ packages. Raw OTU abundances were transformed to relative abundances, which has been suggested as a more appropriate and effective alternative to rarefying^48^. The effect of sample volume on proportion of metazoan taxa, number of unique OTUs, Margalef, and ACE index were tested with one-way analysis of variance (ANOVA). ACE is commonly used as an alpha-diversity measure in HTS investigations that takes into account rare taxa, while the choice of the Margalef index was based on its high sensitivity to sample volume and good capability of discriminating between samples with different alpha-diversity^71^. Homogeneity of variances between groups were tested using Bartlett’s test to examine for all ANOVA analysis. Differences among treatments, i.e sample volumes, in relation to the 100% treatment were tested with a Tukey HSD *posthoc* test for all variables and were considered significant at a p < 0.01.

Community composition was examined by first dividing OTUs into Metazoa, and non-metazoan Eukaryotes groups. The dissimilarity between faunal assemblages in the different sample volumes was analysed by non-metric multidimensional scaling (NMDS), using the Sørensen’s dissimilarity coefficient, and Principal Coordinates Analysis (PCoA) using Bray distances. To test for effects of sample volume on beta-diversity we conducted a PERMANOVA multivariate analysis of variance with the *adonis* function of the *vegan* package^70^. The function PERMDISP was used to test for differences in beta-diversity among sample volumes in relation to the 100% treatment. Additionally, we used metrics to partition beta-diversity to quantify the relative importance of turnover and nestedness in the different sample volumes^72,73^. These authors propose that beta-diversity measured as Sørensen dissimilarity index (βsør) can be separated into dissimilarity as a consequence of turnover, i.e species replacement between sites or samples (βsim), and dissimilarity attributable to nestedness, species loss from sample to sample (βnes). We used the R package *betapart*^74^ for this analysis. Following this, we compared how both components of beta-diversity, turnover and nestedness changed with sample volume in relation to the 100% treatment. All statistical tests were performed on R v 3.3.2.

### Data Accessibility

Data associated with this manuscript is deposited on FigShare (doi:10.6084/m9.figshare.4993667).

## Electronic supplementary material

Supplementary information
