# Supplementary material for: Sample size effects on the assessment of eukaryotic diversity and community structure in aquatic sediments using high-throughput sequencing
Source: Sci Rep. 2018 Aug 6;8:11737. doi: 10.1038/s41598-018-30179-1 (PMC6078945; doi:10.1038/s41598-018-30179-1)
Supplement: Supplementary file 1 — Supplementary information [file 41598_2018_30179_MOESM1_ESM.pdf]

Supplementary information

Sample size effects on the assessment of eukaryotic diversity and community structure in aquatic sediments using high-throughput sequencing

Nascimento, Francisco J.A.[1,4]; Lallias, Delphine[2]; Bik, Holly M[3], Creer, Simon[4]

[1] Department of Ecology, Environment and Plant Sciences, Stockholm University

[2] GABI, INRA, AgroParisTech, Université Paris Saclay, 78350 Jouy-en-Josas, France.

[3]Department of Nematology, University of California Riverside, USA

[4] Molecular Ecology and Fisheries Genetics Laboratory, School of Biological Sciences, Bangor University, LL57 2UW, United Kingdom

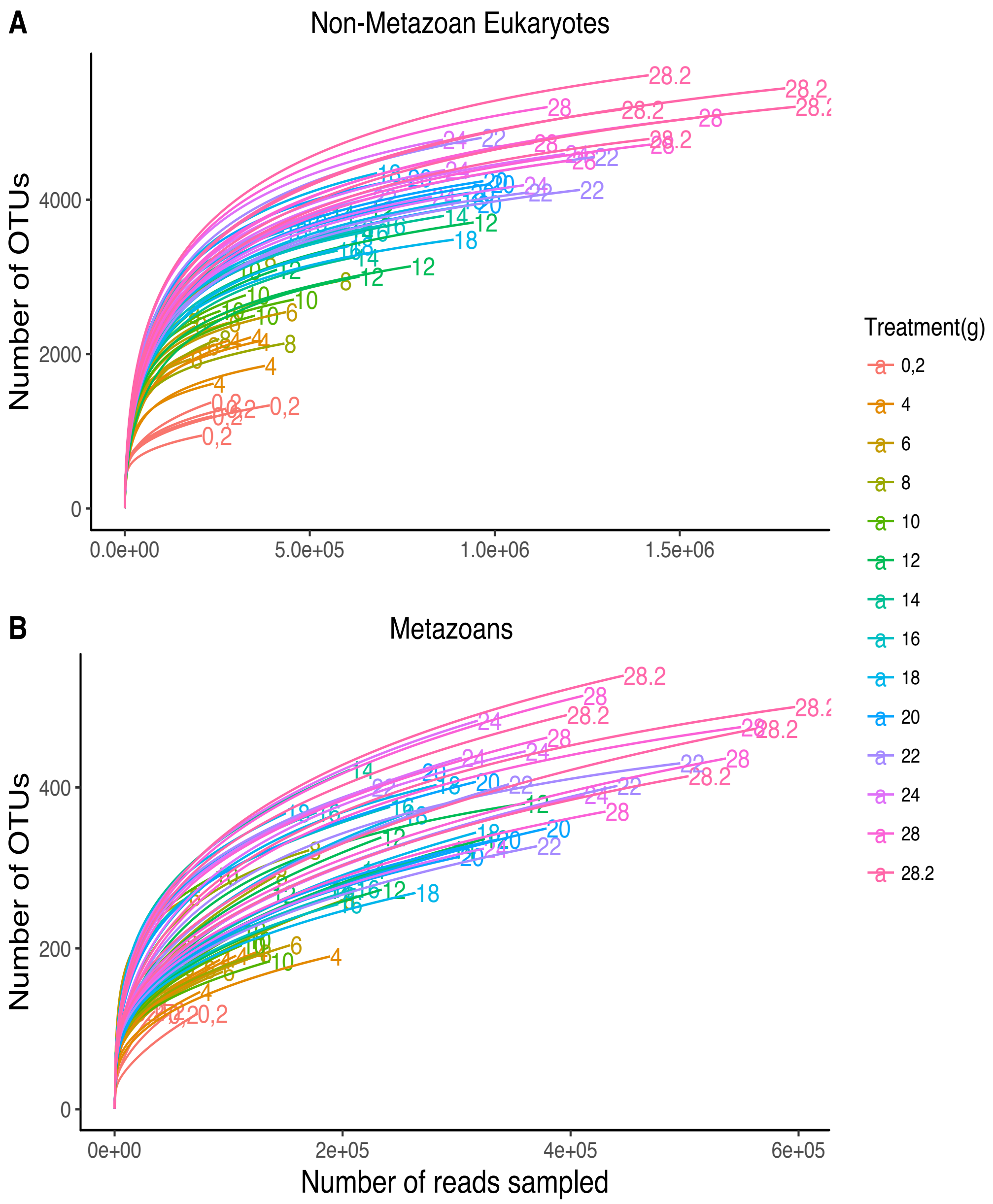

Fig S1- Rarefaction OTU curves for Non-Metazoan Eukaryotes (A) and Metazoans (B) in each sample. **Data represents 96% OTUs.**

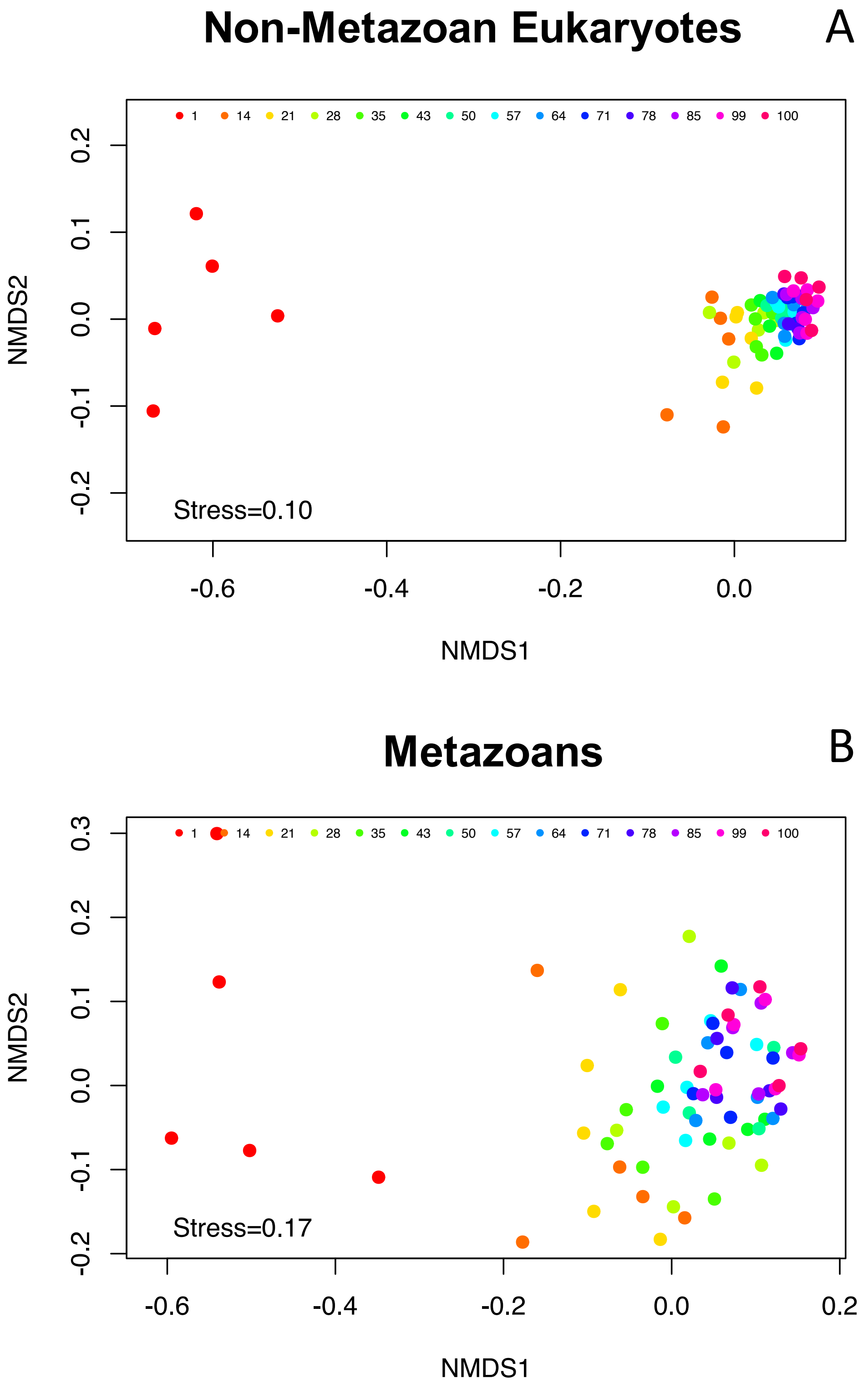

Fig. S2- Complete non-metric multidimensional scaling (NMDS) analysis of the Sørensen beta diversity matrix based on transformed OTU table for Non-Metazoan Eukaryotes (A) and Metazoans (B). **Data represents 96% OTUs.**

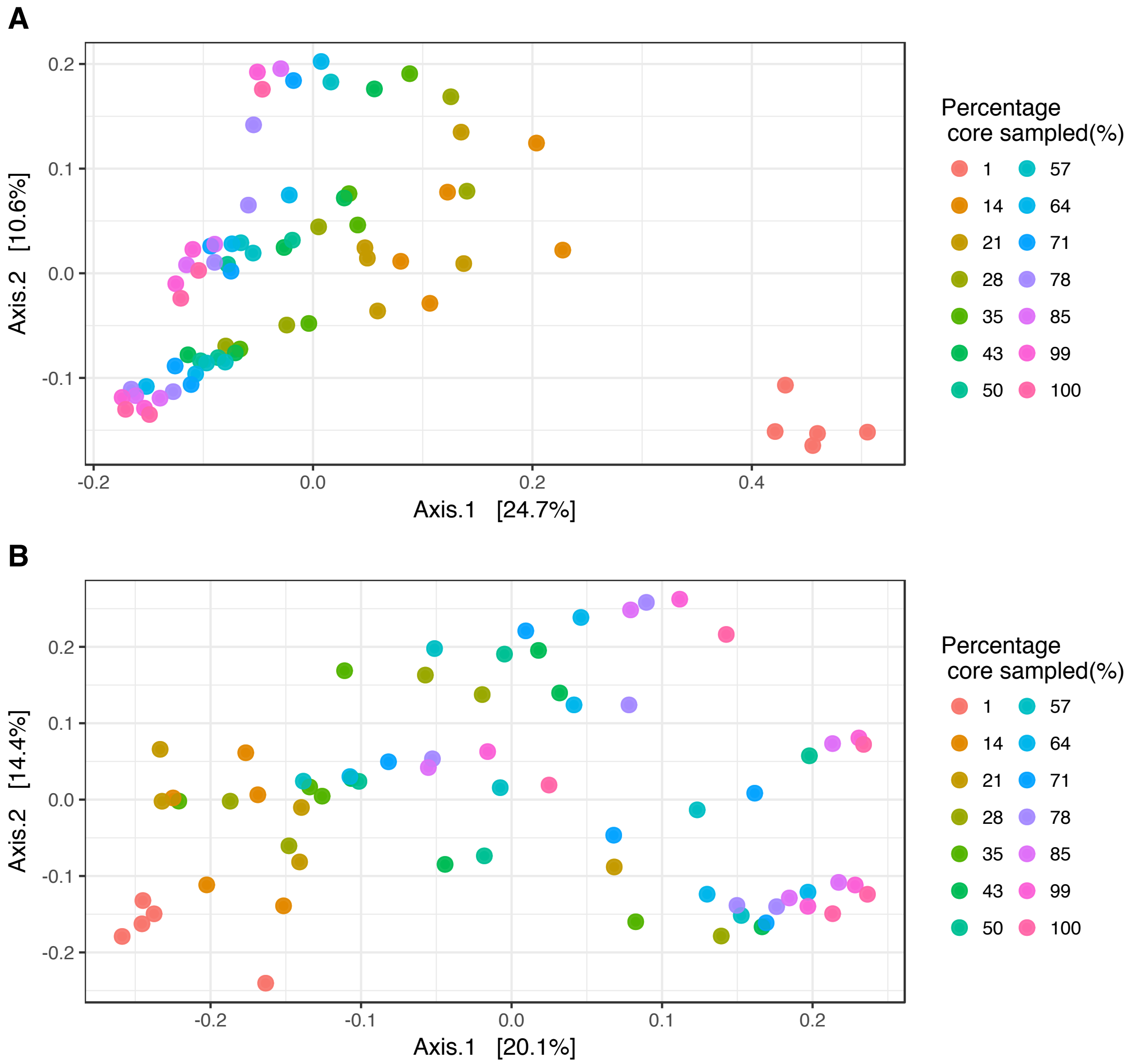

Fig. S3- Principal Coordinates Analysis (PCoA) analysis of the beta diversity matrix based on transformed OTU table for Non-Metazoan Eukaryotes (A) and Metazoans (B). **Data represents 96% OTUs.**

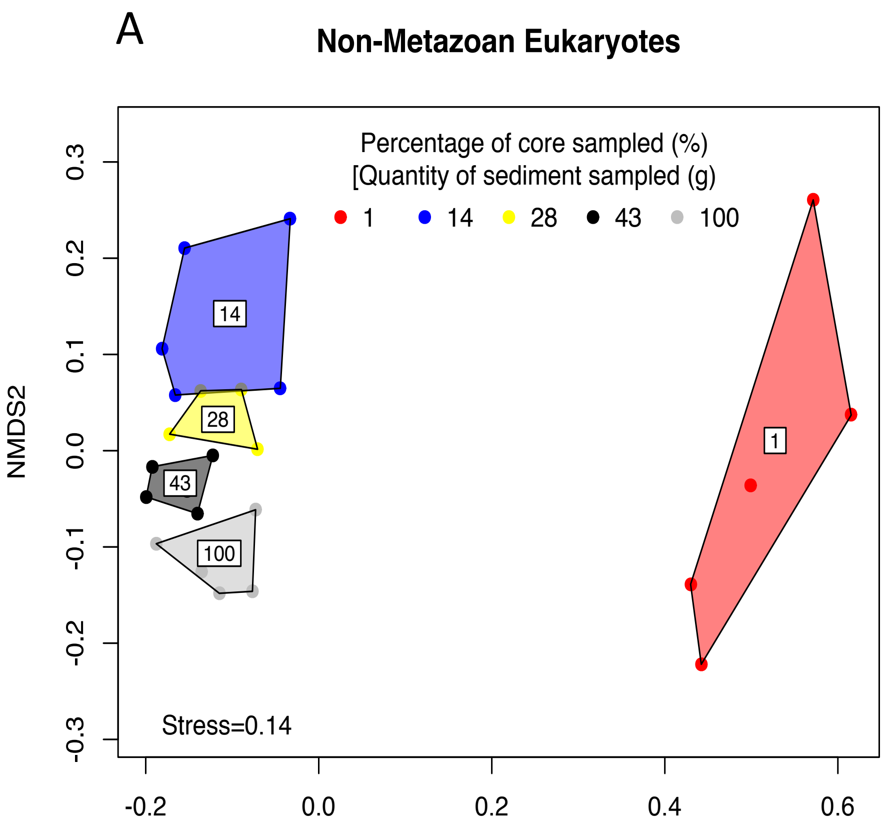
A
Non-Metazoan Eukaryotes
Percentage of core sampled (%)
[Quantity of sediment sampled (g)
1
14
28
43
100
Stress=0.14
NMDS2
-0.3
-0.2
-0.1
0.0
0.1
0.2
0.3
-0.2
0.0
0.2
0.4
0.6

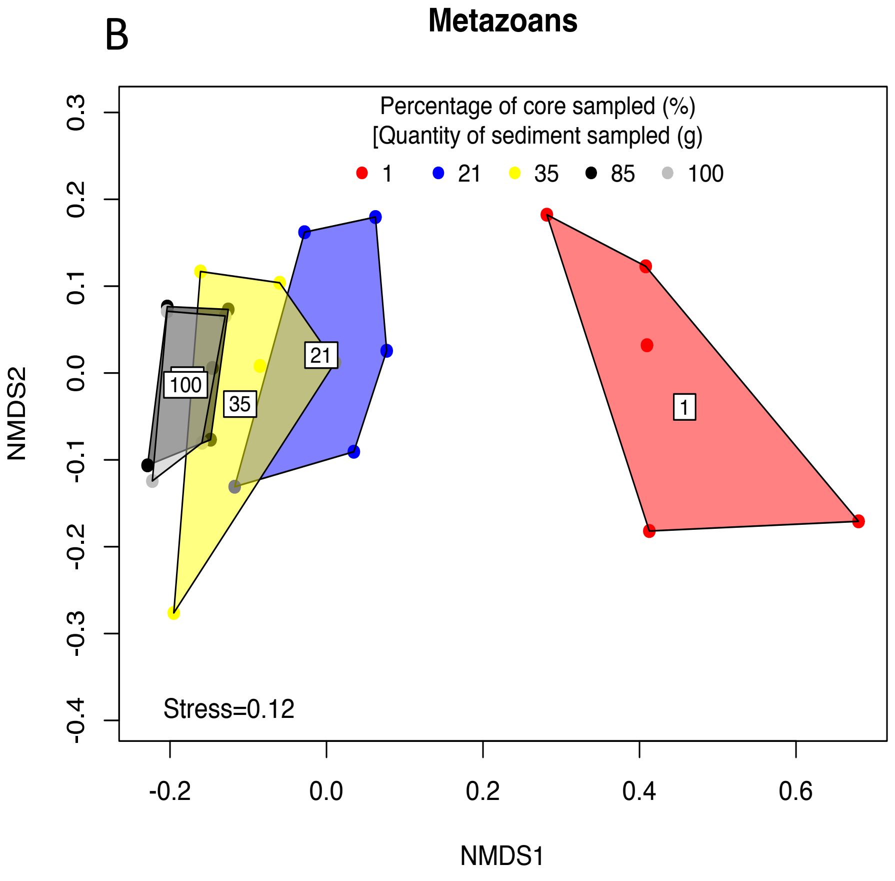
B
Metazoans
Percentage of core sampled (%)
[Quantity of sediment sampled (g)
1
21
35
85
100
Stress=0.12
NMDS2
NMDS1
-0.4
-0.3
-0.2
-0.1
0.0
0.1
0.2
0.3
-0.2
0.0
0.2
0.4
0.6

Fig. S4- Simplified non-metric multidimensional scaling (NMDS) analysis of the Sørensen beta-diversity matrix based on transformed 99% OTU table for Non-Metazoan Eukaryotes (A) and Metazoans (B). Differences between the different treatments and the 100% treatment stopped being significant at 57% of sediment analysed for Non-Metazoan Eukaryotes and in the 64% treatment for Metazoans (PERMANOVA, PERMDISP, see Table S3 in Supplementary information). For better visualization, the same treatments as in Fig 4 in the main text are presented. **Data represents 99% OTUS.**

A

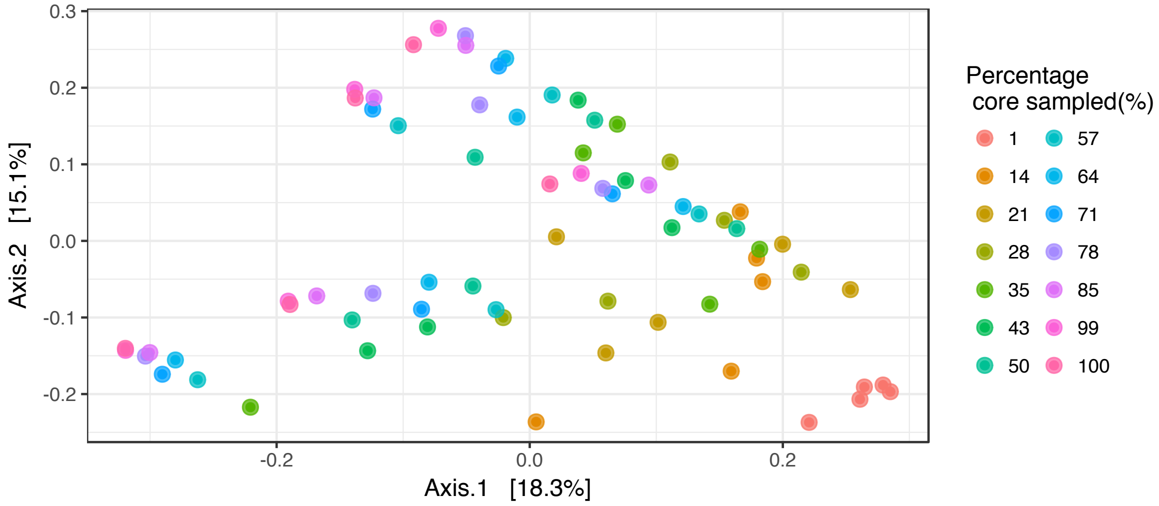

B

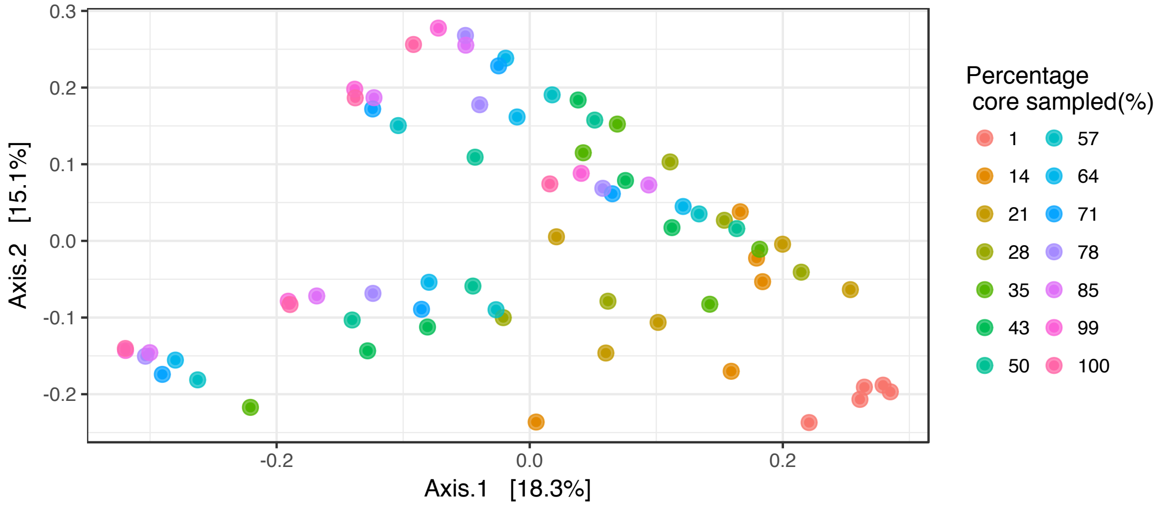

Fig. S5 Principal Coordinates Analysis (PCoA) analysis of the beta diversity matrix based on transformed OTU table for Non-Metazoan Eukaryotes (A) and Metazoans (B). **Data represents 99% OTUs.**

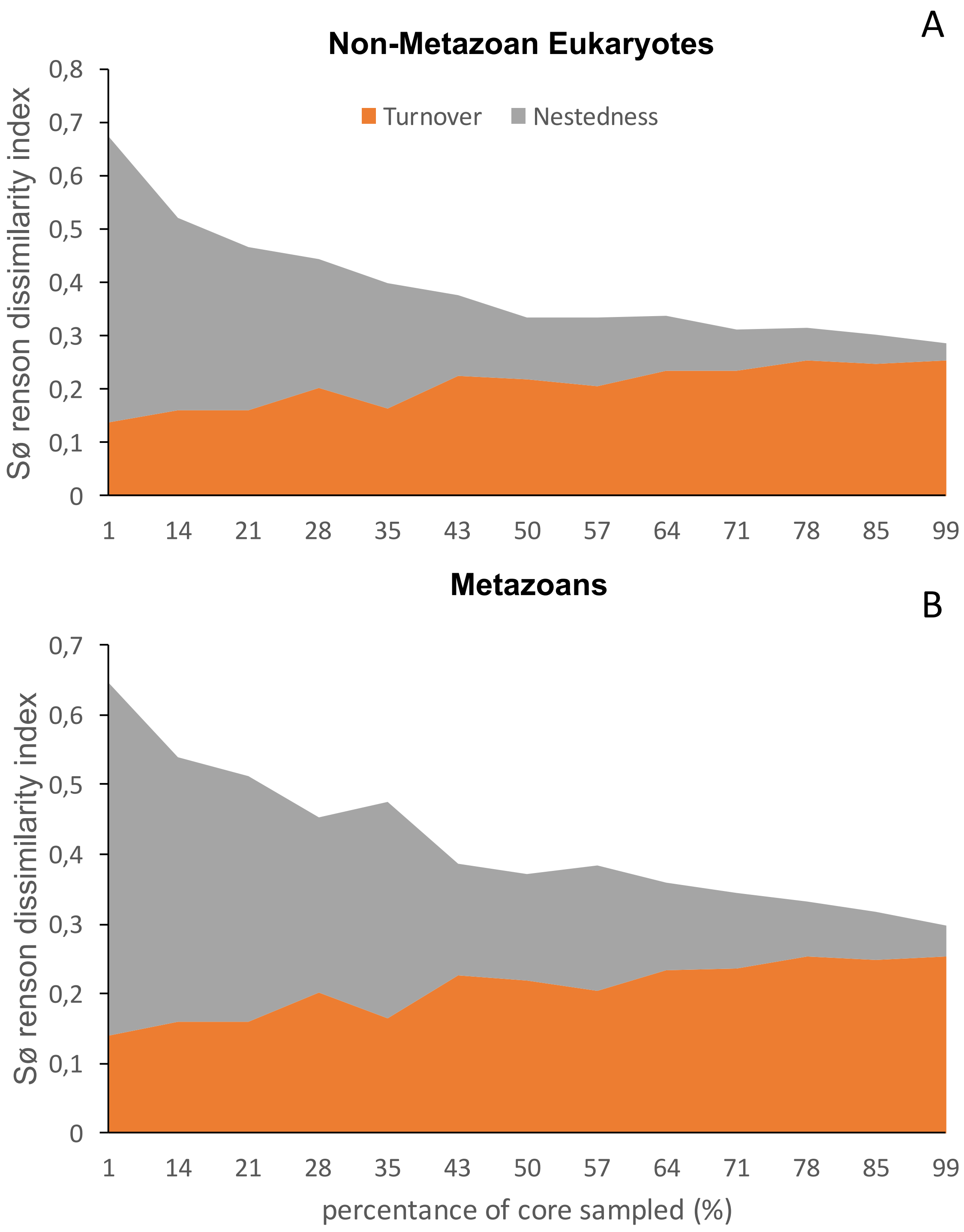

Fig S6- Partitioning of beta-diversity Sorenson dissimilarity index into dissimilarity due to turnover (orange) and nestedness (grey) for Non-Metazoan Eukaryotes (A) and Metazoans (B). **Data represents 96% OTUs.**

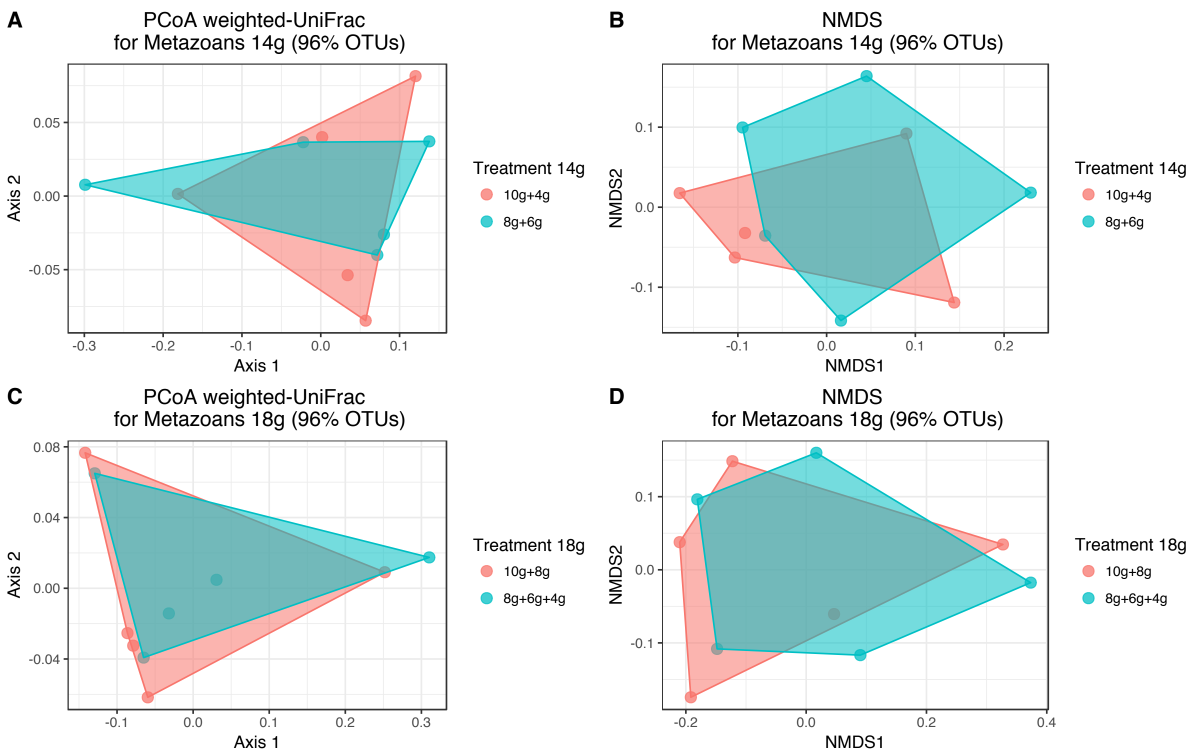

Fig. S7- Weighted PCoA (A and C) and NMDS (B and D) ordinations comparing two different strategies for obtaining 14g (10+4g vs 8+6g) and 18g (10+8g vs 8+6+4g) with the sample volumes used in this study. No statistical differences were seen between the two treatments (PERMANOVA, p=0.53 and p=0.92 for 14g and 18g, respectively)

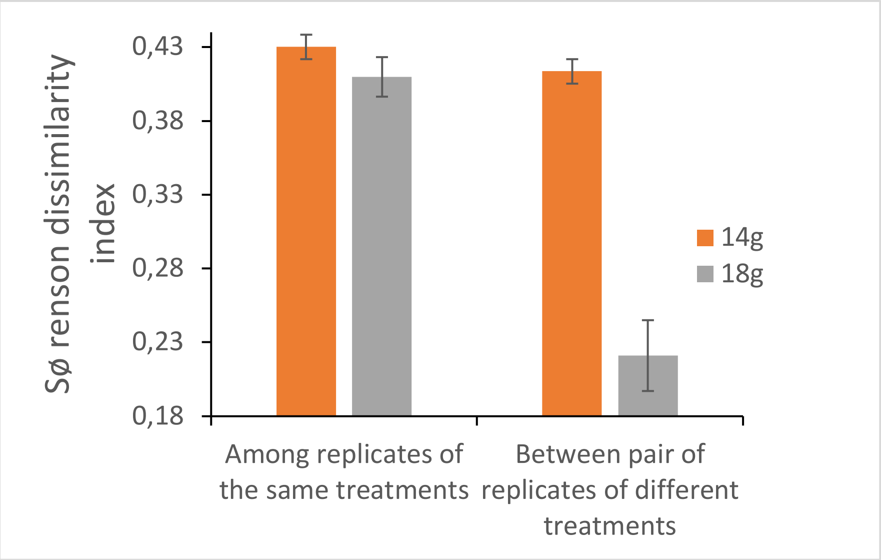

Fig.S8- Average Sørensen dissimilarity index among replicates of the 10g and distance between 10g vs 6+4g of the same replicate (based on data for Metazoans)

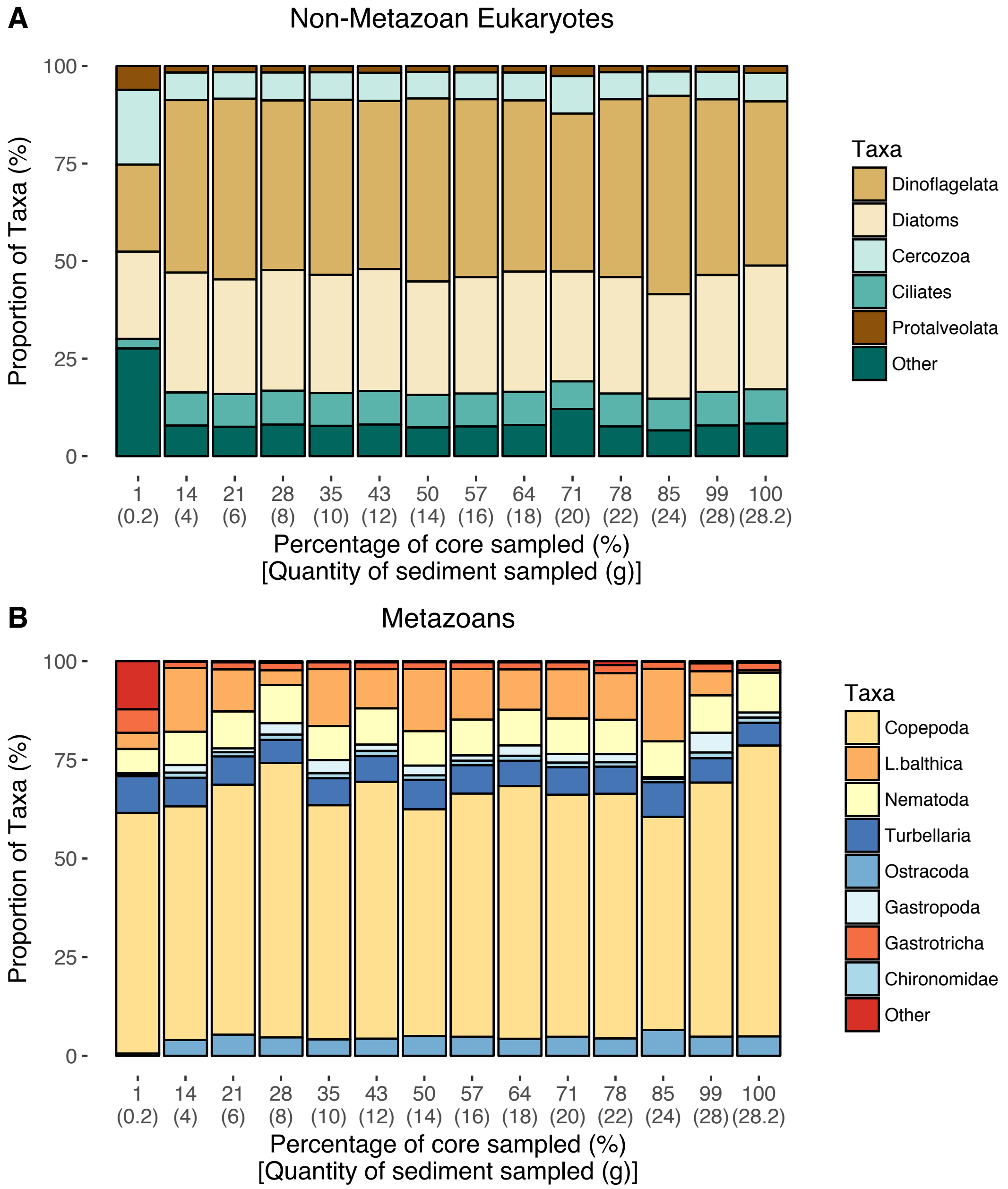

Fig. S9 Proportion of the most abundant taxonomic groups in each treatment: A) Top five most abundant Non-Metazoan Eukaryotes in each treatment; B) Top nine most abundant Metazoan taxa in each treatment. **Data represents 99% OTUs.**

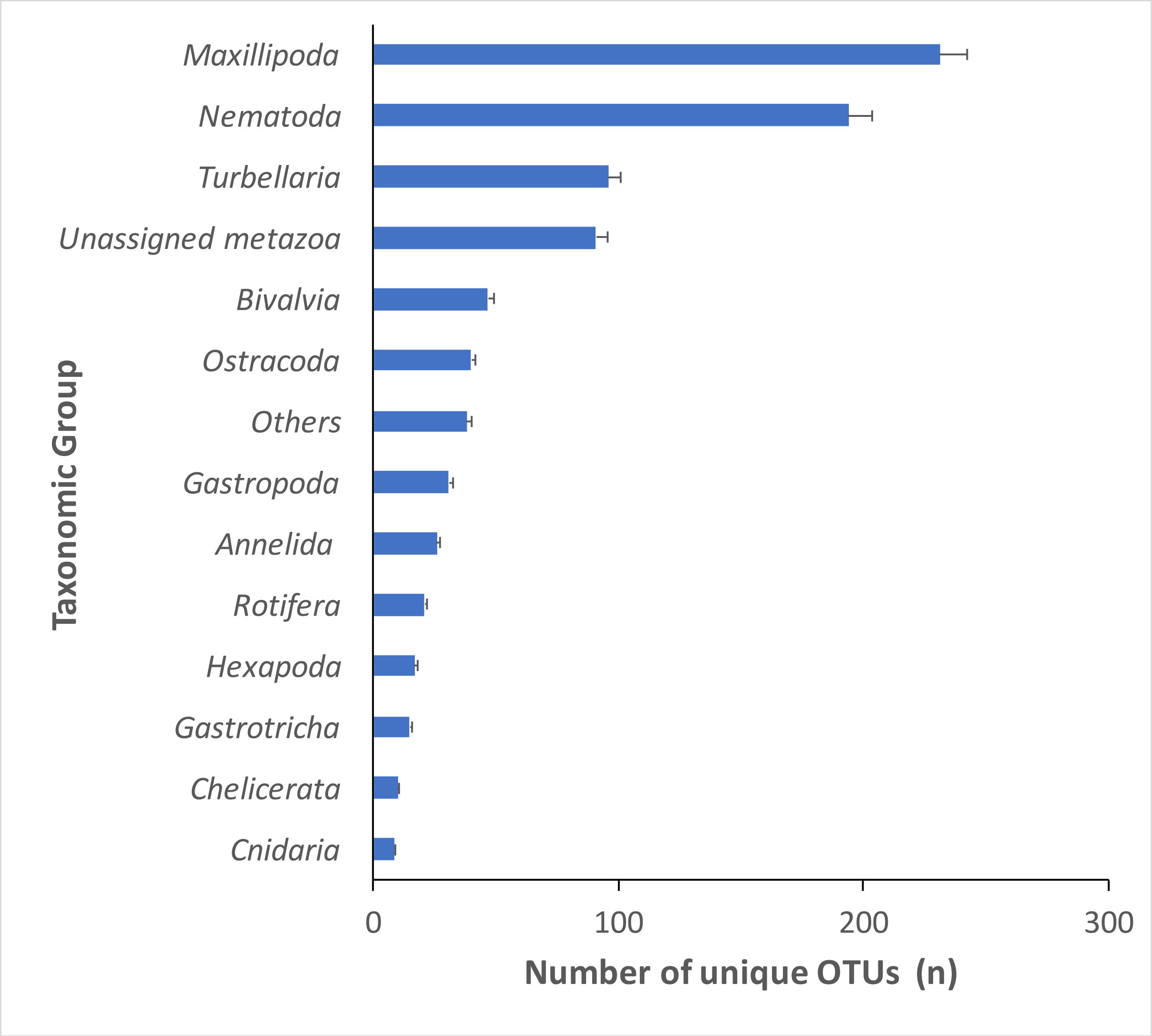

Fig. S10- Average number of unique OTUs per metazoan taxonomic group in the 100% (28.2 g) treatment. **Data represents 96% OTUs.**

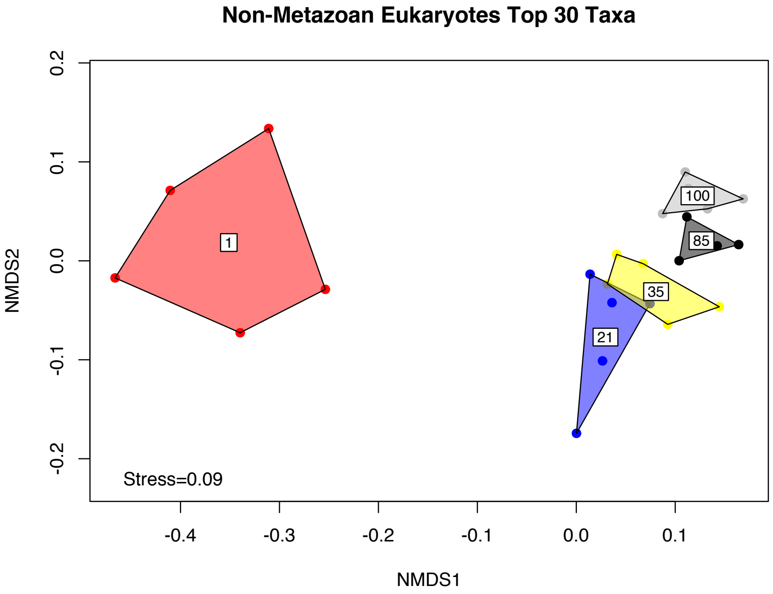

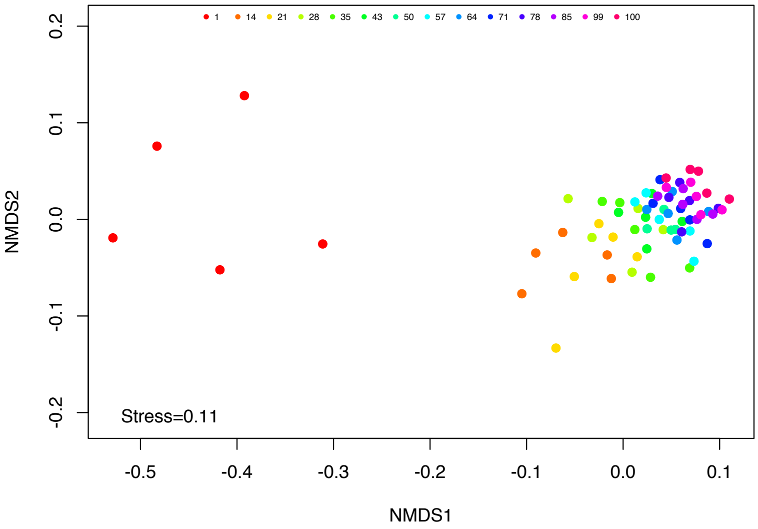

Fig. S11- Simplified (top) and complete (bottom) NMDS of the Sørensen beta diversity matrix based on transformed OTU table of the top 30 Non-Metazoan Eukaryote taxa. **Data based on 96% OTUs**

Table S1- Examples studies that assessed eukaryotic diversity and community structure in aquatic sediments using high-throughput sequencing, with information on sample volumes .

Reference	Target group	Habitat	Methodology	sample volume (g)	main objective
Fonseca *et al.* 2010 [7]	Metazoan meiofauna	Intertidal sandy beach	Isolation of animals from sediment	NA	Assess richness and patterns of diversity of metazoa
Bik *et al.* 2012 [8]	Meiofauna eukaryotes	Abyssal sediments	Both	200	Assess biodiversity of eukaryotes across bathymetric gradients
Stoeck *et al.* 2018 [23]	Benthic ciliates	Marine sediments	eDNA directly from the sediment	6	Impacts of salmon sea cage farming
Zhao&Xu 2016 [24]	Benthic ciliates	Deep-sea sediments	eDNA directly from the sediment	0.3	Evaluate diversity of benthic ciliates from deep-sea hydrothermal vents
Pasulka *et al.* 2016 [25]	Benthic eukaryotes	Methane seep sediments	eDNA directly from the sediment	2	Factors that influence eukaryotic composition and distribution
Volant *et al.* 2016 [26]	Benthic eukaryotes	River sediments	eDNA directly from the sediment	2	Characterize benthic eukaryotic diversity along a pollution gradient
Guardiola *et al.* 2016 [27]	Benthic eukaryotes	Deep-sea sediments	eDNA directly from the sediment	10	Spatial-temporal patterns of diversity in deep-sea sediment communities
Pawlowski *et al.* 2014 [28]	Benthic foraminifera	Muddy sediments	eDNA directly from the sediment	2	Response of foraminifera to environmental gradients
Keeley *et al.* 2018 [29]	Benthic eukaryotes and bacteria	Marine sediments	eDNA directly from the sediment	2	Development of multi-trophic metabarcoding biotic index to assess aquaculture impacts
Laroche *et al.* 2018 [30]	Benthic eukaryotes and bacteria	Marine sediments	eDNA directly from the sediment	2	Impacts of offshore and oil and gas drilling operations
Lejzerowicz *et al.* 2015 [31]	Benthic metazoans	Muddy sediments	eDNA directly from the sediment	2	Compare morphological and eDNA/RNA based inventories of metazoans
Chariton *et al.* 2015 [40]	Benthic eukaryotes	Estuarine sediments	eDNA directly from the sediment	1.5	Benthic eukaryotic diversity of estuaries
Pochon *et al.* 2015[41]	Benthic Foraminifera	Marine sediments	Isolation of animals from sediment	4	Impacts of salmon sea cage farming
Laroche *et al.* 2016 [42]	Benthic Foraminifera	Marine sediments	eDNA directly from the sediment	2	Impacts of offshore and oil and gas drilling operations
Lallias *et al.* 2015 [67]	Metazoan meiofauna	Estuarine sediments	Isolation of animals from sediment	NA	Abiotic factors structuring meifoauna diversity in estuaries
Aguilar *et al.* 2016 [68]	Benthic eukaryotes	Sediment tailing ponds	eDNA directly from the sediment	0.1	Microbial eukaryotic diversity in oil sands tailings ponds

Chariton *et al.* 2014 [69]	Benthic eukaryotes	Muddy sediments	eDNA directly from the sediment	3	Responses of benthic eukaryotes to exposure to triclosan
Fonseca et al. 2017 [70]	Metazoan meiofauna	Antarctic sediments	Isolation of animals from sediment	NA	Investigate biodiversity in Antarctic sediments
Lecroq *et al.* 2011 [71]	Benthic foraminifera	Deep-sea sediments	eDNA directly from the sediment	0.5 (5g total)	Examine the richness of benthic deep-sea foraminifera
Lanzén *et al.* 2016 [72]	Benthic eukaryotes	Marine sediments	eDNA directly from the sediment	5	Environmental monitoring of offshore oil-drilling activities
Bahdury&Austen 2010 [73]	Benthic nematodes	Surface sandy sediments	eDNA directly from the sediment	0.5	Test existing taxonomy databases
Smith *et al.* 2016 [74]	Dinoflagellate	Algal beds	eDNA directly from the sediment	0.5	Biodiversity assessment of benthic-epiphytic dinoflagellates

Table S2- Previous studies on the effects of sample size on diversity with details on the investigated taxa, used sample sizes, and directionality of the effects found

**Reference**	**Target group**	**Samples sizes tested (g)**	**Marker**	**Effects on diversity**
Kang & Mills 2006 [17]	Soil bacteria	0.01, 0.1,0.25, 1, 10	16S	Effects of sample size but no difference between 0.25 and 10g
Penton *et al.* 2016	Soil bacteria and fungi	0.25, 1,5, 10	16S and 28S	Effects of sample size. 10g with highest estimates of eveness, diversity and richness
Ellingsǿe & Johnsen 2002 [29]	Soil bacteria	0.01, 0.1, 1.0, 10	16S	Effects of sample size but no difference between 1 and 10g
Brannock & Halanych 2015 [12]	Meiofauna	0.3,5, 10 and animals elutriated from 200 and 400	18S	No effect of sample size on alpha-diversity; differences in beta-diversity between elutriated and non-elutriated samples

Table S3- Summary of statistical tests comparing the different treatments with the treatment comprising 100% of the sampled area (based on 99% OTUs). One-way parametric analysis of variance ANOVA was used to test for the effect of sample volume. P values smaller than 0.05 indicate a significant effect of sample volume. Pairwise comparisons between the 100% treatment and the other 13 treatments were performed with the Tukey HSD test. Effects of sample volume on beta-diversity differences among treatments were tested with PERMANOVA. Differences between the 100% treatment and the other 13 treatments for beta-diversity was tested with PERMDISP. The treatment with the highest sample volume still different from the treatment comprising 100% of the sampled area in each of the variable is presented in the last column of the table. **Data represents 99% OTUs.**

**Variable**	**Community**	**ANOVA F statistics**	**ANOVA p value ANOVA**	**Highest % of core sampled significantly different from the 100% treatment**
**Percentage of metazoan taxa**	NA	3.1	< 0.001	1% (0.2g)
**Number of unique OTUs**	Non-Metazoan Eukaryotes	28	< 0.001	78% (22g)
	Metazoans	23	< 0.001	64% (18g)
**Margalef index**	Non-Metazoan Eukaryotes	29	< 0.001	71% (20g)
	Metazoans	24	< 0.001	57% (16g)
**Chao1 richness**	Non-Metazoan Eukaryotes	55	< 0.001	78% (22g)
	Metazoans	15	< 0.001	57% (16g)

		**PERMANOVA F Statists**	**PERMANOVA p value**	**Lowest % of core sampled significantly different from the 100% treatment**
**Beta-Diversity**	Non-Metazoan Eukaryotes	1.6	<0.001	57% (16g)
	Metazoans	1.2	0.024	64% (18g)

Table S4- Number of sequence reads for each of the 25 analysed samples before and after demultiplexing and sequence quality filtering

Replicate	Sample volume	Raw sequences reads	N of demultiplexed and filtered sequence reads
0.2g-A	0.2g	234 451	229 408
0.2g-B	0.2g	392 771	384 917
0.2g-C	0.2g	273 938	268 099
0.2g-D	0.2g	237 462	232 721
0.2g-E	0.2g	209 164	205 334
4g-A	4g	361 511	355 290
4g-B	4g	342 117	336 490
4g-C	4g	283 206	278 231
4g-D	4g	240 372	236 532
4g-E	4g	379 050	373 392
6g-A	6g	281 583	276 898
6g-B	6g	190 847	187 496
6g-C	6g	178 641	175 437
6g-D	6g	436 053	429 110
6g-E	6g	223 693	219 840
8g-A	8g	582 388	572017
8g-B	8g	432 078	425 029
8g-C	8g	378 646	371 463
8g-D	8g	171 791	168 672
8g-E	8g	256 068	252 215
10g-A	10g	327 693	321 773
10g-B	10g	457 717	449 949
10g-C	10g	304 376	298 608
10g-D	10g	259 511	255 074
10g-E	10g	352 061	345 941

Table S5- Total number of sequences for each of the additional sample volumes based on combined samples (see Methods)

Replicate	Replicate combination	Total number of sequence reads
12g-A	8g-A + 4g-A	9.4E+05
12g-B	8g-B + 4g-B	7.7E+05
12g-C	8g-C + 4g-C	6.6E+05
12g-D	8g-D + 4g-D	4.1E+05
12g-E	8g-E + 4g-E	6.3E+05
14g-A	8g-A + 6g-A	8.6E+05
14g-B	8g-B + 6g-B	6.2E+05
14g-C	8g-C + 6g-C	5.6E+05
14g-D	8g-D + 6g-D	6.1E+05
14g-E	8g-E + 6g-E	4.8E+05
16g-A	10g-A + 6g-A	6.1E+05
16g-B	10g-B + 6g-B	6.5E+05
16g-C	10g-C + 6g-C	4.8E+05
16g-D	10g-D + 6g-D	6.9E+05
16g-E	10g-E + 6g-E	5.8E+05
18g-A	10g-A + 8g-A	9.1E+05
18g-B	10g-B + 8g-B	8.9E+05
18g-C	10g-C + 8g-C	6.8E+05
18g-D	10g-D + 8g-D	4.3E+05
18g-E	10g-E + 8g-E	6.1E+05
20g-A	10g-A + 6g-A + 4g-A	9.7E+05
20g-B	10g-B + 6g-B + 4g-B	9.9E+05
20g-C	10g-C + 6g-C + 4g-C	7.7E+05
20g-D	10g-D + 6g-D + 4g-D	9.3E+05
20g-E	10g-E + 6g-E + 4g-E	9.5E+05
22g-A	10g-A + 8g-A + 4g-A	1.3E+06
22g-B	10g-B + 8g-B + 4g-B	1.2E+06
22g-C	10g-C + 8g-C + 4g-C	9.6E+05
22g-D	10g-D + 8g-D + 4g-D	6.7E+05
22g-E	10g-E + 8g-E + 4g-E	1.1E+06
24g-A	10g-A + 8g-A + 6g-A	1.2E+06
24g-B	10g-B + 8g-B + 6g-B	1.1E+06
24g-C	10g-C + 8g-C + 6g-C	8.6E+05

24g-D	10g-D + 8g-D + 6g-D	8.7E+05
24g-E	10g-E + 8g-E + 6g-E	8.3E+05
28g-A	10g-A + 8g-A + 6g-A + 4g-A	1.6E+06
28g-B	10g-B + 8g-B + 6g-B + 4g-B	1.4E+06
28g-C	10g-C + 8g-C + 6g-C + 4g-C	1.1E+06
28g-D	10g-D + 8g-D + 6g-D + 4g-D	1.1E+06
28g-E	10g-E + 8g-E + 6g-E + 4g-E	1.2E+06
28.2g-A	10g-A + 8g-A + 6g-A + 4g-A + 0.2g-A	1.8E+06
28.2g-B	10g-B + 8g-B + 6g-B + 4g-B + 0.2g-B	1.8E+06
28.2g-C	10g-C + 8g-C + 6g-C + 4g-C + 0.2g-C	1.4E+06
28.2g-D	10g-D + 8g-D + 6g-D + 4g-D + 0.2g-D	1.3E+06
28.2g-E	10g-E + 8g-E + 6g-E + 4g-E + 0.2g-E	1.4E+06
